# Compensation of a Second Harmonic Wave Included in an Incident Ultrasonic Wave for the Precise Measurement of the Acoustic Nonlinearity Parameter

**DOI:** 10.3390/s21093203

**Published:** 2021-05-05

**Authors:** Dong-Gi Song, Sungho Choi, Taehyeon Kim, Kyung-Young Jhang

**Affiliations:** 1 Department of Mechanical Convergence Engineering, Hanyang University, Seoul 04763, Korea; dgsong@hanyang.ac.kr; 2 LANL-JBNU Engineering Institute-Korea, Jeonbuk National University, Jeollabuk-do 54896, Korea; schoi@jbnu.ac.kr; 3 Radiation and Decommissioning Laboratory, KHNP-CRI, Daejeon 34101, Korea; taehyeon.kim@khnp.co.kr; 4 School of Mechanical Engineering, Hanyang University, Seoul 04763, Korea

**Keywords:** absolute acoustic nonlinearity parameter, nonlinear ultrasonic technique, incident second harmonic wave

## Abstract

The incident second harmonic wave is a problematic issue for the precise measurement of the acoustic nonlinearity parameter. This paper proposes a compensation method to remove the effect of the incident second harmonic component in the measurement of the absolute acoustic nonlinearity parameter using the calibration method. For this, the second harmonic component detected by the receiving transducer is considered as the sum of the component due to material nonlinearity and the component included in the incident signal and a numerical calculation model is developed as a function of the propagation distance. In the model, the factors related to the material nonlinear parameter and the magnitude of the incident second harmonic component are unknown and these are determined by finding a value that best matches the experimental data according to the change in the propagation distance; compensation for the incident second harmonic component is then achieved. The case where the phase of the second harmonic wave due to material nonlinearity is opposite to that of the fundamental wave is also considered. To verify the validity of the proposed method, fused silica and aluminum alloy Al6061-T6 specimens with different thicknesses corresponding to the propagation distance are tested. The experimental results show that the nonlinear parameters changed significantly according to the propagation distance before compensation but were very stable after compensation. Additionally, the average values of the nonlinear parameter are 11.04 in the fused silica, which is within the literature value range (10.1 to 12.4), and that for the Al6061-T6 is 6.59, which is close to the literature value range (4.5 to 6.12).

## 1. Introduction

The nonlinear ultrasonic technique has been researched as a nondestructive method to diagnose material degradation [1,2,3]. For this, the acoustic nonlinearity parameter *β*, defined by the displacement amplitudes of the fundamental and second harmonic waves, is generally employed [4]. Furthermore, contact transducers have been frequently used for transmitting and receiving an ultrasonic wave because they have a higher signal-to-noise ratio and it is simple to set up the measurement system [5]. However, they detect the ultrasonic wave in the form of an electric signal, the amplitude of which is not a displacement signal. Therefore, the pre-measurement of a transfer function to convert the electric amplitude into the displacement amplitude should be performed; this is called the calibration method [6,7].

Meanwhile, the diffraction effect due to the finite source size and the attenuation effect from the material absorption and scattering interrupt the accurate measurement of the acoustic nonlinearity parameter [8,9,10]. For this, Jeong et al. [11] proposed attenuation and diffraction correction methods with experimental results for thick, solid aluminum samples, which showed that the proposed methods reduced the measurement error of the acoustic nonlinearity parameter. However, there is still a problem to be solved, which is that the second harmonic wave is included in the signal incident to the test specimen. Such an incident second harmonic wave may occur in the transducer, in the contact interface between the transducer and the specimen under test and in the signal-generating devices, which is generally at a level that cannot be ignored in the measurement of the acoustic nonlinear parameter using high-power signals. This effect has been called system nonlinearity or source nonlinearity [12,13]. Therefore, for the correct measurement of the acoustic nonlinearity parameter, it is necessary to remove the amplitude of the second harmonic wave included in the incident wave so that only the amplitude of the second harmonic wave generated by the acoustic nonlinearity of the material can be detected.

In this regard, Kim et al. [12] reported that a large error of about 43% occurs in the measurement result when the incident wave already contains an extra second harmonic component. Although there are few studies related to the compensation of the incident second harmonic wave, Torello et al. [13] proposed a method for the measurement of the nonlinear Rayleigh wave in which the magnitude of the second harmonic wave in the received signal was regarded as the sum of the incident component and the component due to material nonlinearity. The magnitude of each component according to the change in propagation distance was expressed as a sound field analysis model and by finding the model parameters that best matched the experimental result, the magnitude of the incident second harmonic component was specified. However, as this method is for Rayleigh waves and can be applied only to relative measurements, it cannot be applied to the calibration method that uses longitudinal waves for absolute acoustic nonlinearity parameter measurements.

Therefore, in this study we propose a method for the compensation of the incident second harmonic wave in the measurement of the absolute acoustic nonlinearity parameter using the calibration method. The basic idea is similar to Torello’s work in the aspect that it compares the sound field analysis model and the experimental results for the magnitude of the received second harmonic wave according to the change of the propagation distance. However, within the scope of this study, the sound field was modeled for longitudinal waves and the magnitude of the second harmonic wave was modeled not with the signal amplitude but with the absolute displacement amplitude. For this, the linear wave propagation model using the distributed point source method was adopted for the fundamental wave and the incident second harmonic component. Meanwhile, the second harmonic wave generated by material nonlinearity was calculated using the model of Ingenito and Williams [14] in which the second harmonic waves emitted from each point of the virtual source field (with an amplitude proportional to the square of the local fundamental amplitude) propagate as a linear wave.

We also considered the case where the phase of the second harmonic due to material nonlinearity was in phase with the fundamental wave as well as the case where it was out of phase. The second harmonic displacement amplitudes according to the propagation distance were then measured from tests on several specimens of different thicknesses. Finally, the amplitude of the incident second harmonic wave was estimated by finding the theoretical model that best fitted the experimental result and it was removed so that the second harmonic amplitude generated by only material nonlinearity could be obtained.

For experimental verification, the proposed method was applied to specimens of fused silica and Al6061-T6, whose phases of the second harmonics caused by material nonlinearity are opposite to each other [15]. To vary the propagation distance in both materials, several specimens with different thicknesses were prepared. The attenuation effect was not considered because it was negligible when considering the short propagation distance in the experiment and the low attenuation coefficient in both materials [16,17].

## 2. Absolute Acoustic Nonlinearity Parameter

The absolute acoustic nonlinearity parameter (β) is defined by the squared displacement amplitude of the fundamental wave ($A_{1}^{2}$) and the displacement amplitude of the second harmonic wave ($A_{2}$), the propagation distance (*x*) and the wave number (*k*) as follows [7,18]:

(1) $$\beta =\frac{{8A}_{2}}{k^{2}{xA}_{1}^{2}}.$$

A method for the reliable measurement of the acoustic nonlinearity parameter is to use the linearity of $A_{2}$ and $A_{1}^{2}$ (measure $A_{2}$ while changing $A_{1}$) or the linearity of $A_{2}$ and *x* (measure $A_{2}$ while changing *x*) when the other parameters are fixed [19]. However, neither of these methods were suitable for this study. In general, to change the fundamental wave amplitude, the input power must be controlled. If the second harmonic component is included in the input signal, it is difficult to separate the input second harmonic wave and the second harmonic wave from the material because it also changes with the fundamental wave component according to the change of the input power. Therefore, this method was chosen here in which, as the input power is kept constant, the amplitudes of the incident fundamental and second harmonic waves are kept constant and only the amplitude of the second harmonic component changes according to the change of propagation distance due to the acoustic nonlinearity of the medium. Of course, the diffraction effect according to the change of the propagation distance is also considered with this. However, as the transmission signals of longitudinal waves are used, specimens of different thickness must be prepared in order to change the propagation distance, as was done in this study.

## 3. Numerical Calculation of the Ultrasonic Wave Field

The distributed point source method developed by Kundu et al. [20,21] is a semi-analytical technique to compute the ultrasonic pressure field based on the Rayleigh–Sommerfeld integral. The transducer surface and the target field are regarded as an assemblage of distributed small points.

**Figure 1:**
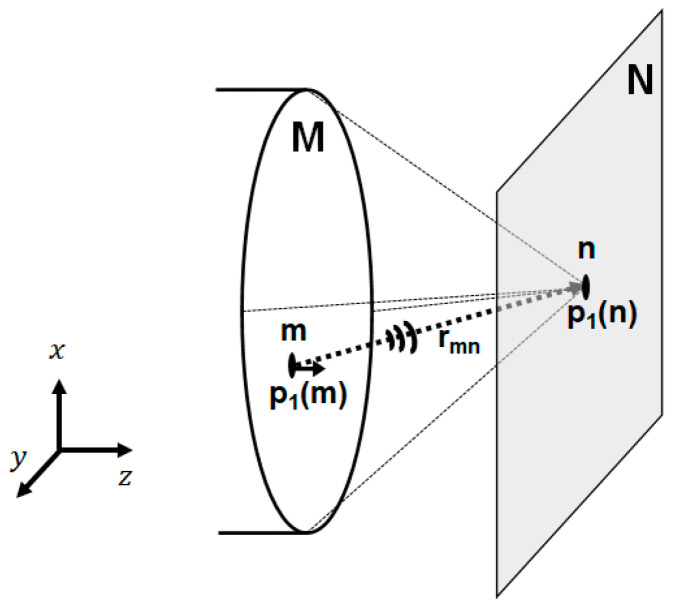
Basic concept of the distributed point source method.

As shown in Figure 1, the ultrasonic wave generated at a certain small point (*m*) on the transducer surface (*M*) reaches a certain small point (*n*) in the target field (*N*). The ultrasonic wave at point *n* can then be determined by the summation of the ultrasonic waves from all of the small points on the transducer surface (*M*). Thus, the pressure $p_{1}\left(n\right)$ can be expressed by pressure $p_{1}\left(m\right)$ at the point *m* on the transducer as follows [20]:

(2) $$p_{1}\left(n\right)=\sum_{m=1}^{M}p_{1}\left(m\right)G\left(n,m\right)\;\mathrm{with}\;G\left(n,m\right)=\frac{e^{{\mathrm{ikr}}_{\mathrm{mn}}}\;}{r_{\mathrm{mn}}}$$

where the G(n,m) terms are Green’s functions, which describe the propagation from the source point *m* to the target point *n* and $r_{\mathrm{mn}}$ is the distance between point *m* and point *n*. In this study we assumed that the attenuation was negligible so the absorption attenuation term is not included. The pressure profile on the transducer surface was obtained from the velocity profile as follows:

(3) $$p_{1}\left(m\right){=i\rho \omega v}_{z}\left(m\right)$$

where ω is the angular frequency, ρ is the density and $v_{z}\left(m\right)$ is the velocity profile on the transducer surface. The initial boundary condition was adopted as the velocity profile of the transducer surface calculated from the multi-Gaussian beam model with 25 orders when the center velocity of the transducer was given [22,23,24]. The center velocity will be later referred to as the initial velocity $v_{\mathrm{initial}}$. Using this velocity profile, the pressure $p_{1}\left(m\right)$ can be determined and then the pressure field can be calculated.

**Figure 2:**
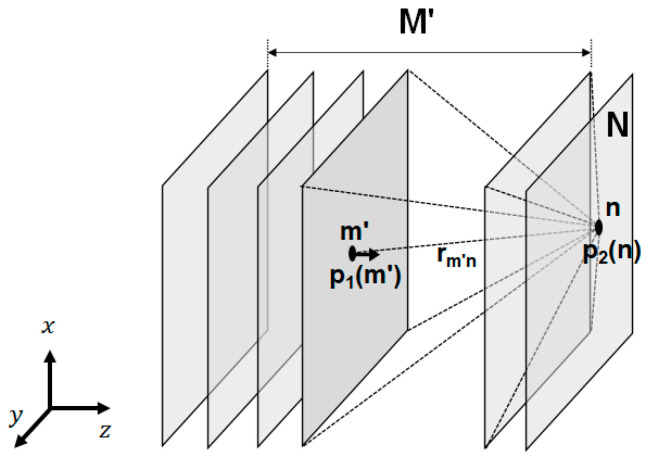
Model of the second harmonic pressure field calculation method.

Meanwhile, the nonlinear generation in the quasi-linear approximation was treated as the emission of a second-order wave from each point in the domain of the linear wave propagation, as shown in Figure 2. This can be visualized as a field of virtual sources $M^{\prime }$; the pressure of each source being proportional to the square of the local first-order pressure. In this study, the model of Ingenito and Williams [14,25,26] was used to calculate the second-order field at a given point in a sound beam as follows:

(4) $$p_{2_{\mathrm{material}}}\left(n\right)=\frac{k^{2}\;\beta }{{4\pi \rho c}^{2}}\sum_{m^{\prime }=1}^{M^{\prime }}p_{1}^{2}\left(m^{\prime }\right)G\left({n,m}^{\prime }\right)\;\mathrm{with}\;G\left({n,m}^{\prime }\right)=\frac{e^{2ikr_{m^{\prime }n}}}{r_{m^{\prime }n}}$$

where $p_{2_{\mathrm{material}}}\left(n\right)$ is the second-order pressure at a point *n* in the target field *N*, $p_{1}{(m}^{\prime })$ is the local first-order pressure associated with a virtual source, β is the acoustic nonlinearity parameter and *c* is the wave velocity.

The computational process consists of two steps. The first step is to calculate the linear field of pressure $p_{1}\left(n\right)$ at any point using Equation (2). This was carried out for all points in a slice of the region parallel to the transducer plane. These first-order amplitudes were then squared and, using the appropriate Green’s functions, propagated onto the target point. This was then repeated for all slices of the region and the contributions from all of the slices were summed.

**Figure 3:**
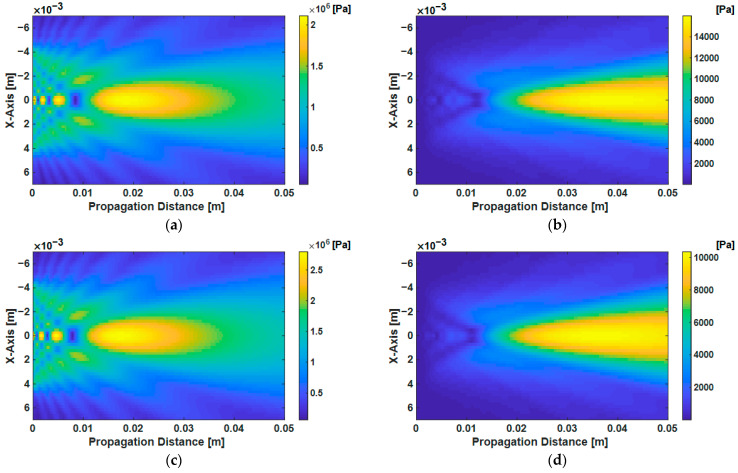
Numerical calculation results of the ultrasonic pressure field of fundamental wave $p_{1}\left(n\right)$ calculated using Equation (2), and second harmonic wave $p_{2_{material}}\left(n\right)$ using Equation (4) with = 0.1 m/s at 5 MHz driving frequency: (**a**) fundamental wave and (**b**) second harmonic wave in fused silica; (**c**) fundamental wave and (**d**) second harmonic wave in the Al6061-T6.

Figure 3a,b show the calculated ultrasonic pressure fields of the fundamental and second harmonic waves in fused silica as the *x-z* cross-sectional distribution, the material parameters of which are $\rho =2.17\;\mathrm{kg}/m^{3}$, c=5980 m/s and β=10. Figure 3c,d show them in the Al6061-T6, the material parameters of which are $\rho =2.69\;\mathrm{kg}/m^{3}$, c=6400 m/s and β=6 [27]. The diameter of the transducer is 0.375 in and the driving frequency is 5 MHz and $v_{\mathrm{initial}}$ was given as 0.1 m/s.

**Figure 4:**
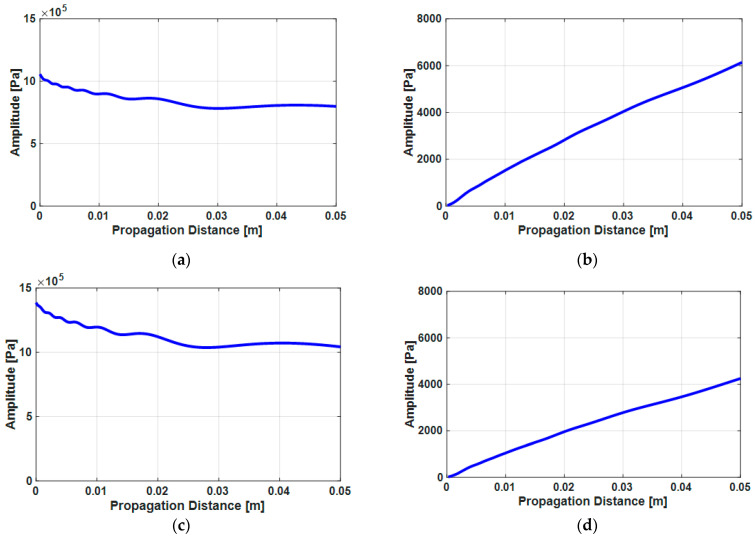
The received pressure amplitude on the receiver as a function of the distance to the transmitter, obtained from the average of the sound pressure in Figure 3: (**a**) the received fundamental amplitude and (**b**) the received second harmonic amplitude in the fused silica; (**c**) the received fundamental amplitude and (**d**) the received second harmonic amplitude in the Al6061-T6.

Here, the received pressure amplitude can be calculated as the average pressure of all of the points belonging to the receiving transducer surface. Figure 4 shows the received pressure amplitude of the fused silica and the Al6061-T6 when the receiver transducer is the same size as the transmitter transducer (0.375 in). Figure 4a,b are the received fundamental and second harmonic amplitudes in the fused silica and c and d are those in the Al6061-T6. The fundamental amplitude decreases due to the diffraction effect but the second harmonic amplitude increases according to the propagation distance because of the growth of the second harmonic component by the material even though it also includes the diffraction effect.

Now we consider the case in which a second harmonic component is included in the incident wave. Here this component will be referred to as the incident second harmonic wave. As this wave only needs to be considered as a linear wave propagation, the pressure of the second harmonic wave at any point in the target field can be expressed in a form similar to Equation (2) as follows:

(5) $$p_{2_{\mathrm{incident}}}\left(n\right)=\sum_{m=1}^{M}p_{2_{\mathrm{incident}}}\left(m\right)G(n,m)$$

where $p_{2_{\mathrm{incident}}}\left(m\right)$ is the initial pressure of the incident second harmonic wave on the transducer. In the calculation, for convenience, $p_{2_{\mathrm{incident}}}\left(m\right)$ is assumed to be proportional to the square of $p_{1}\left(m\right)$ as follows:

(6) $$p_{2_{\mathrm{incident}}}\left(m\right){=\beta }_{\mathrm{incident}}p_{1}^{2}\left(m\right)$$

where $\beta_{\mathrm{incident}}$ is the incident nonlinearity proportional factor. The total second harmonic pressure $p_{2}\left(n\right)$ is the summation of the second harmonic pressure by material nonlinearity (Equation (4)) and the incident second harmonic pressure (Equation (5)) as follows [13]:

(7) $$p_{2}\left(n\right){=p}_{2_{\mathrm{material}}}\left(n\right){+p}_{2_{\mathrm{incident}}}\left(n\right).$$

**Figure 5:**
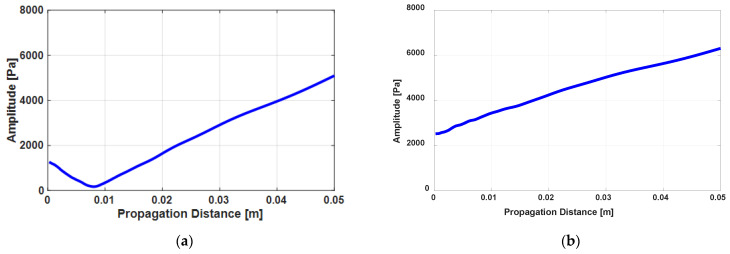
Received pressure amplitude of the second harmonic wave calculated using Equation (7) at the frequency 10 MHz including the incident second harmonic wave in two cases: (**a**) the opposite phase (fused silica) and (**b**) the same phase (Al6061-T6).

Meanwhile, in general, the second harmonic wave by material nonlinearity has the same phase as the incident second harmonic wave; however, there is a special case in which their phases are opposite to each other such as in fused silica. To consider this, the phase of the incident second harmonic wave can be changed in the calculation. Figure 5 shows the received pressure amplitude of the second harmonic wave when $\beta_{\mathrm{incident}}$ is −1.0 × 10^−13^ in fused silica and 1.0 × 10^−13^ in the Al6061-T6. The calculated incident second harmonic pressure amplitude at z = 0 is 1.24 kPa in Figure 5a and 2.49 kPa in Figure 5b.

**Figure 6:**
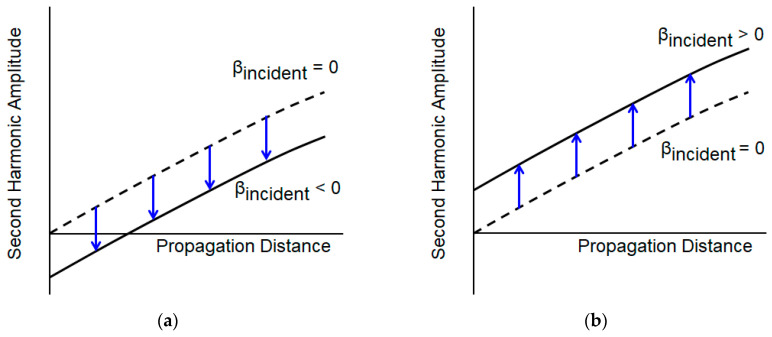
The trend of the second harmonic amplitude according to the propagation distance when the incident second harmonic wave exists in (**a**) fused silica and (**b**) Al6061-T6.

We can see that Figure 5a,b appear differently. In most of the literature, as the acoustic nonlinearity parameter is expressed as absolute value, the sign is not considered. However, it can be positive or negative depending on the material. In fused silica, the sign of the nonlinear parameter is negative while that for Al6061-T6 is positive [15,16]. However, in this study, for convenience, the signs of the nonlinear parameters in both materials were set as positive and instead, the sign of $\beta_{\mathrm{incident}}$ for fused silica was taken as negative. Figure 6 explains the effect of this setting in more detail in which Figure 6a corresponds with Figure 5a and Figure 6b corresponds with Figure 5b. In the case of fused silica, the amplitude of the second harmonic generated by the material nonlinearity should increase as the propagation distance increases, as shown by the dotted line in Figure 6a. However, if there exists an incident harmonic component whose phase is opposite, it will shift the dotted line downwards. However, as the magnitude of the second harmonic is measured in an absolute value, the result appears as shown in Figure 5a. On the other hand, in the Al6061-T6, as shown in Figure 6b, the incident component is simply added to shift the dotted line upwards of which the pattern is the same as in Figure 5b.

Finally, because the displacement amplitude is proportional to the pressure amplitude, the fundamental displacement $A_{1}\left(z\right)$ received at the propagation distance z can be expressed by displacement and pressure as follows:

(8) $$A_{1}\left(z\right)=\frac{1}{\rho c\omega }\left|p_{1}\left(z\right)\right|.$$

In a similar way, the second harmonic displacement amplitude $A_{2}\left(z\right)$ received at the propagation distance *z* can be expressed as follows:

(9) $$A_{2}\left(z\right)=\left|A_{2_{\mathrm{material}}}\left(z\right){+A}_{2_{\mathrm{incident}}}\left(z\right)\right|=\frac{1}{\rho c\omega }\left|p_{2_{\mathrm{material}}}\left(z\right){+p}_{2_{\mathrm{incident}}}\left(z\right)\right|$$

where $A_{2_{\mathrm{incident}}}\left(z\right)$ and $A_{2_{\mathrm{material}}}\left(z\right)$ represent the received second harmonic displacement amplitudes of the component in which the incident second harmonic wave propagates and the component generated by the material nonlinearity, respectively. Similarly, $p_{2_{\mathrm{incident}}}\left(z\right)$ and $p_{2_{\mathrm{material}}}\left(z\right)$ are the received second harmonic pressure amplitudes. The proportional factors 1/ρcω are 2.45 × 10^−15^ m/Pa for fused silica and 1.85 × 10^−15^ m/Pa for Al6061-T6 at 5 MHz and 1.23 × 10^−15^ m/Pa for fused silica and 0.924 × 10^−15^ m/Pa for Al6061-T6 at 10 MHz.

**Figure 7:**
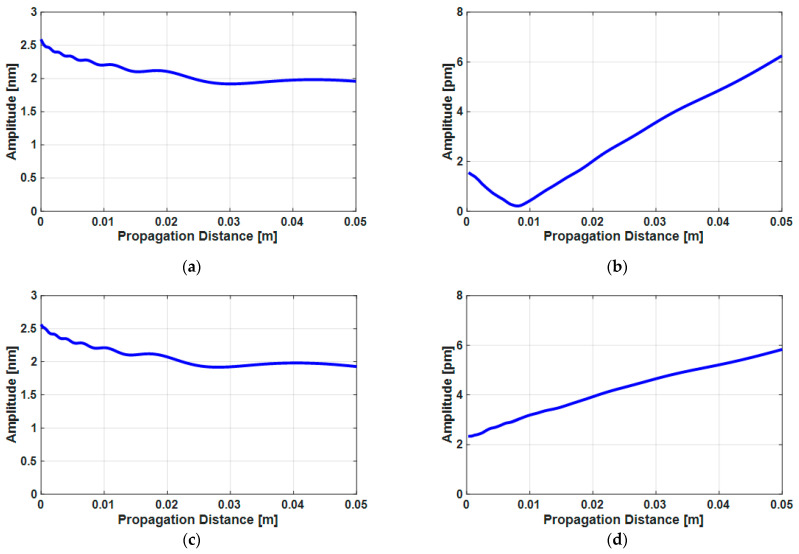
The numerical calculation results of the received displacement amplitude of fundamental wave $A_{1}\left(z\right)$ calculated using Equation (8) at a frequency of 5 MHz and the second harmonic wave $A_{2}\left(z\right)$ calculated using Equation (9) at the frequency of 10 MHz according to the propagation distance: (**a**) the fundamental wave and (**b**) the second harmonic wave in fused silica and (**c**) the fundamental wave and (**d**) the second harmonic wave in the Al6061-T6.

Figure 7 shows the numerical calculation results of the fundamental and second harmonic displacement amplitudes. The incident displacement of the fundamental wave at z = 0 is 2.55 nm in fused silica and 2.52 nm in the Al6061-T6 and that of the second harmonic wave is 1.52 pm in fused silica and 2.30 pm in the Al6061-T6.

## 4. Estimation and Correction Methods of Incident Second Harmonic Amplitude

**Figure 8:**
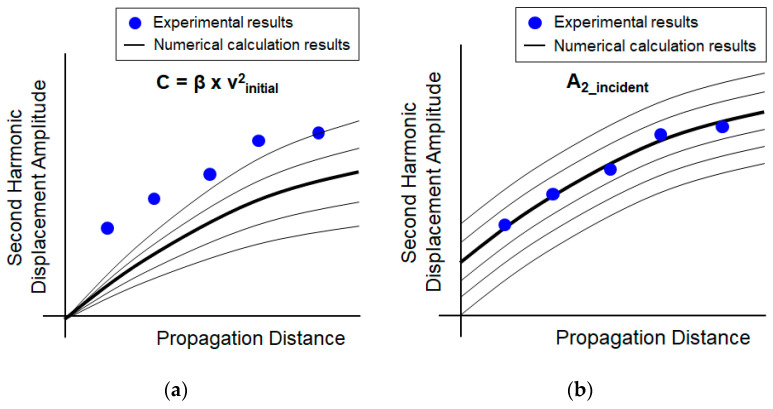
Estimation of second harmonic displacement amplitude ($A_{2}\left(z\right)$) as a function of the propagation distance *z* calculated using Equation (9), also indicating the relationship to Figure 7d; (**a**) $A_{2}\left(z\right)$ for $p_{2_{\mathrm{incident}}}(z)=0$ and (**b**) $A_{2}\left(z\right)$ for $p_{2_{\mathrm{incident}}}\left(z\right)$ not equal to 0 in the same phase.

To obtain an accurate acoustic nonlinearity parameter, β, the displacement amplitude of the incident second harmonic wave, $A_{2_{\mathrm{incident}}}\left(z\right)$, must be removed from the displacement amplitude of the received second harmonic, $A_{2}\left(z\right)$, in Equation (9). For this, we propose a method of fitting the aforementioned theoretical calculation model to the change in the amplitude of the second harmonic wave according to the change in propagation distance obtained experimentally. This method consists of two steps and its schematic is shown in Figure 8.

First, for the case where the incident second harmonic does not exist ($p_{2_{\mathrm{incident}}}(z)=0$), calculate the pressure amplitude of the second harmonic according to the propagation distance, as shown in Figure 4b or Figure 4d, by using Equation (4) with the material to be tested and the experimental conditions and then, using Equation (9), calculate the displacement amplitude. In this case, the nonlinear parameter β and the initial velocity $v_{\mathrm{initial}}$ are unknown so they remain as variables. Here, because the curve trend changes proportionally according to the product of β and squared $v_{\mathrm{initial}}$, the unified coefficient *C* defined in Equation (10) is used in the estimation process:

(10) $${C=\beta \times v}_{\mathrm{initial}}.$$

Figure 8a shows the calculation results for several different coefficients and the coefficient that best matches the experimental data indicated by the circular dots with the trend of change over the propagation distance is selected. The bold line indicates the best matched curve.

Next, for the case where the incident second harmonic exists, calculate the displacement amplitude of the second harmonic wave according to the propagation distance as shown in Figure 7d by using Equation (9). In this case, the incident second harmonic amplitude is unknown so it remains as a variable. Figure 8b shows the calculation results for several different incident second harmonic amplitudes and the amplitude that best matched the curve is selected. The incident second harmonic amplitude is then determined by the value of the starting point of the matched curve.

Although this explanation has been divided into two steps, they are not actually separated; the calculations are done all at once. That is, the coefficient and the amplitude of the incident second harmonic are set as variables and the values that minimize the difference between the experimental results are found. The least squares method is applied here.

**Figure 9:**
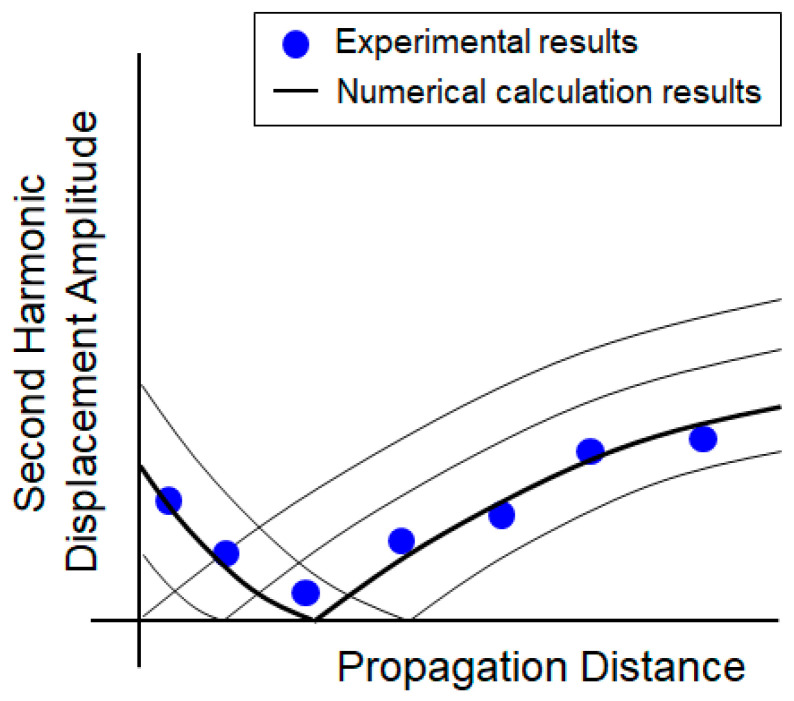
Estimation of the second harmonic displacement amplitude ($A_{2}\left(z\right)$) for $p_{2_{\mathrm{incident}}}\left(z\right)$ not equal to 0 in a negative phase, also indicating a relationship to Figure 7b.

On the other hand, if the incident second harmonic is opposite to the phase with the second harmonic generated by the material, the amplitude of the received second harmonic initially tends to decrease and then increase again from a certain propagation distance, as shown in Figure 9. If this phenomenon occurs in the experimental data, the phase of the incident second harmonic and the second harmonic generated from the material are opposite so a theoretical calculation model with the phase of the incident second harmonic as negative, as shown in Figure 7b, should be used.

**Figure 10:**
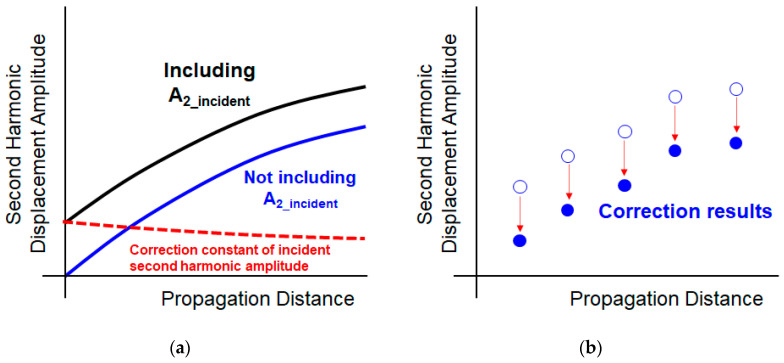
Correction process of the incident second harmonic amplitude ($A_{2}\left(z\right)$): (**a**) calculation of the correction factor and (**b**) correction of the experimental data.

Finally, the correction factor $A_{2_{\mathrm{incident}}}\left(z\right)$ is obtained from the difference between the two theoretical models that, respectively, include and do not include the incident second harmonic component, which is obtained in the estimation process, as shown Figure 10a. This component is subtracted from the experimental data to obtain the final second harmonic displacement amplitude, as shown in Figure 10b.

## 5. Experiment

**Figure 11:**
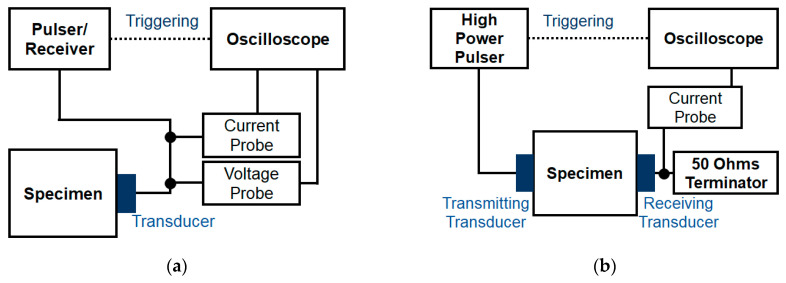
Experimental setup for (**a**) calibration and (**b**) harmonic measurement.

Figure 11 shows the experimental setup for the measurement of the absolute acoustic nonlinearity parameter, which consists of two steps: calibration and harmonic measurement. In the calibration, the transfer function to convert the received electric signal into a displacement signal is measured for the transducer to be used as a receiver in the harmonic measurement. Dace et al. [7] interpreted that the conversion efficiency for electric to acoustic power is equal to that for acoustic to electric power and derived the transfer function from the relationship between the input signal and the received first echo signal in the pulse-echo method, which is defined as follows:

(11) $$\left|H(\omega )\right|=\sqrt{\frac{\left|I_{in}\left(\omega \right)\right|\left(\frac{\left|V_{out}\left(\omega \right)\right|}{\left|I_{out}\left(\omega \right)\right|}\right)+\left|V_{in}\left(\omega \right)\right|}{{2\omega }^{2}\rho ca\left|I_{out}\left(\omega \right)\right|}}$$

where ($\left|V_{in}\left(\omega \right)\right|$ and $\left|I_{\mathrm{in}}\left(\omega \right)\right|$) are the magnitude spectra of the voltage and the current signal of the input and ($\left|V_{out}\left(\omega \right)\right|$ and $\left|I_{out}\left(\omega \right)\right|$) are those of the first echo signal and *a* is the area of the transducer surface.

When the calibration measurement is finished, the transmitting transducer is attached onto the specimen while maintaining contact with the receiving transducer. The current signal from the receiving transducer is then detected (this is called harmonic measurement) and the amplitudes of the fundamental and second harmonic components are analyzed in the frequency domain. Finally, the displacement amplitude can be calculated from the transfer function and the received current signal as Equation (12):

(12) $$\left|A(\omega )\right|=\left|H(\omega )\right|\left|I(\omega )\right|$$

where $\left|A(\omega )\right|$ is the displacement amplitude and $\left|I(\omega )\right|$ is the spectrum of the received current signal in the harmonic measurement.

In the calibration measurement, the broadband signal is generally employed to obtain the transfer function over a wide frequency range that includes the fundamental and second harmonic frequencies. For this, a pulser-receiver (5072PR, Olympus) that could drive the spike pulse up to 400 V was employed. The current and voltage probes were set between the pulser-receiver and the transducer to measure the input signal and first echo signal. For the harmonic measurement, a high-power pulser (RAM-5000-M4, RITEC Inc.) to drive a 20-cycle tone-burst signal with 5 MHz was employed. The current probe was set between the receiving transducer and a 50 ohms terminator to collect the ultrasonic signal. In both measurements, a voltage probe (P5100A, Tektronix) with a 500 MHz bandwidth up to 2.5 kV and a current probe (CP030, Teledyne LeCroy) with a 50 MHz bandwidth up to 30 A were used. An oscilloscope (HDO4034A, Teledyne LeCroy) with a 12 bit resolution and 10 GS/s collected all of the signals from the probes in the condition of full-scale height over 80%. A 5 MHz transducer was used as the transmitter with a 3.20 to 6.90 MHz bandwidth and a 10 MHz transducer with a 4.95 to 11.90 MHz bandwidth was used as the receiver to sensitively detect second harmonic waves; both were 0.375 in in size. Fused silica and Al6061-T6 specimens were prepared with various thicknesses (fused silica: 5, 10, 15, 20, 25, 30, 40 mm; Al6061-T6: 10, 15, 20, 25, 30, 35, 40 mm). Note that in all experiments, a contact pressure of about 300 kPa was applied to the transducer using a specially designed pneumatic system to maintain the same contact condition between the transducer and the specimen [28].

**Figure 12:**
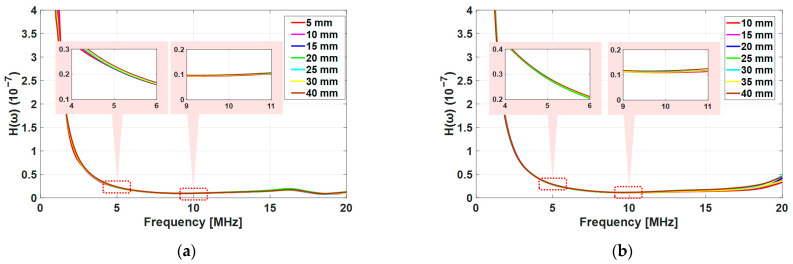
Transfer functions of the receiving transducer ($\left|H(\omega )\right|$) following from the calibration measurement after diffraction correction: (**a**) fused silica and (**b**) Al6061-T6.

Figure 12 shows the results of the transfer functions for the fused silica and the Al6061-T6 in the calibration measurement including the diffraction corrections. The deviation of the transfer function was under 3.64% for the fused silica and under 1.37% for the Al6061-T6 in the frequency range of interest. These results confirmed that the contact state of the transducer in the experiment was kept constant and both materials had a low attenuation. Note that as the contact state of the transducer can affect the magnitude of the incident harmonic wave, it was necessary to keep it constant for each specimen in the experiment.

**Figure 13:**
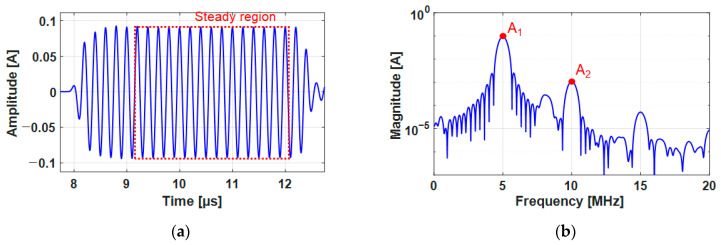
An example of signal obtained from the harmonic measurement; (**a**) received tone-burst signal from the current probe (*I(t)*) and the selected steady region and (**b**) fast Fourier transform result (*I(f)*) of the selected steady signal.

Figure 13a shows the received signal from the current probe in the harmonic measurement where the static component was removed beforehand. The signal processing was performed for only the signal in the steady region where the amplitude remained constant. Figure 13b is the fast Fourier transform of the selected signal in which the second harmonic component was clearly detected. The fundamental and second harmonic displacement amplitudes were calculated using the transfer function at 5 and 10 MHz and the current signal amplitudes at 5 and 10 MHz in the received signal using Equation (12). Meanwhile, we could see third and fourth harmonics at 15 MHz and 20 MHz, respectively, which could be also generated by the nonlinear characteristics of the material but they were out of our interest.

## 6. Estimation and Correction Results of Incident Second Harmonic Amplitude from the Experimental Results

**Figure 14:**
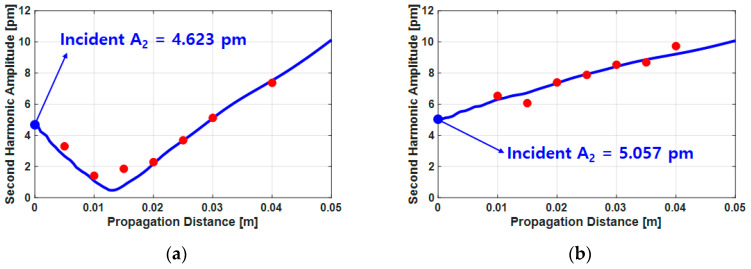
Results of the displacement amplitude of the second harmonic wave according to propagation distance in (**a**) fused silica and (**b**) Al6061-T6.

Figure 14 shows the experimental results of the second harmonic displacement amplitude according to the propagation distance for the fused silica and the Al6061-T6; the estimation process was conducted as described in Section 4. The best matched trends were selected by changing the parameter $\beta_{\mathrm{incident}}$ and the coefficient *C* based on the numerical calculation results in Figure 7b,d and the initial second harmonic displacement amplitudes were determined. It is worth noting here that it is not necessary to know the parameter and the coefficient. The blue lines in Figure 14 are final estimations. In the fused silica, the incident second harmonic displacement amplitude was estimated to be 4.623 pm and the displacement amplitude of the second harmonic decreased until the propagation distance of about 0.013 m and increased thereafter. It was confirmed that incident second harmonic wave had a phase opposite to that of the second harmonic wave from the material nonlinearity of the fused silica. In the Al6061-T6, the incident second harmonic amplitude was 5.057 pm and the displacement of the second harmonic wave increased with respect to the propagation distance.

**Figure 15:**
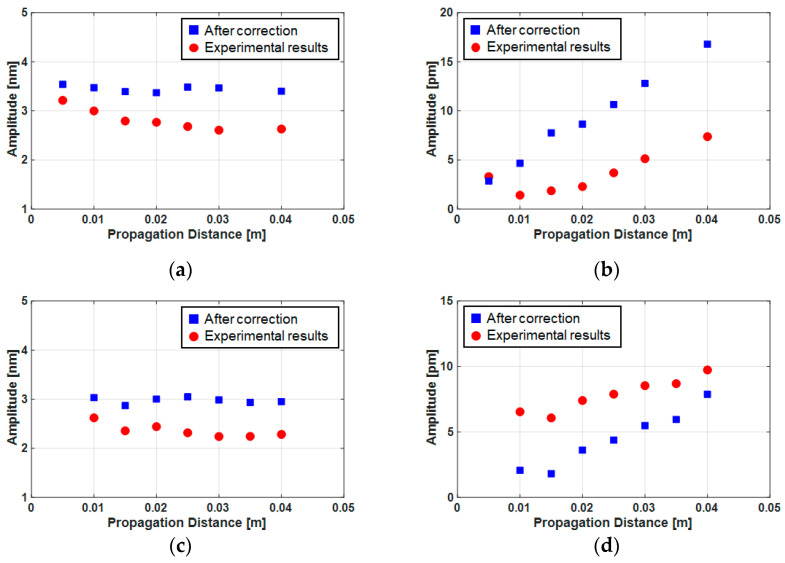
Results of the measurement and correction of the fundamental ($A_{1}\left(z\right)$) and the second harmonic ($A_{2}\left(z\right)$) displacement amplitudes: (**a**) fundamental component of fused silica, (**b**) second harmonic amplitude of fused silica, (**c**) fundamental component of Al6061-T6 and (**d**) second harmonic amplitude of Al6061-T6.

Figure 15 shows the correction results of the fundamental and second harmonic amplitudes; a and b are for the fused silica specimens and c and d are for the Al6061-T6 specimens. In Figure 15a,c, for fundamental amplitudes, the red dots represent the experimental data and the blue squares are the diffraction corrections. We could see that the correction results were almost constant regardless of the propagation distance. In Figure 15b,d for the second harmonic amplitudes, the red dots represent the experimental data and the blue squares are after the correction of the diffraction and the incident second harmonic component. In the case of fused silica, the amplitude of the second harmonic generated by the material nonlinearity was subtracted by the incident component whose phase was opposite and the detected amplitude decreased. Therefore, if we compensated for this, it increased to its original amplitude. On the other hand, in the Al6061-T6, as the phase of the second harmonic generated by the material nonlinearity and the incident component were the same, the detected magnitude increased. Therefore, it became smaller after the correction.

**Figure 16:**
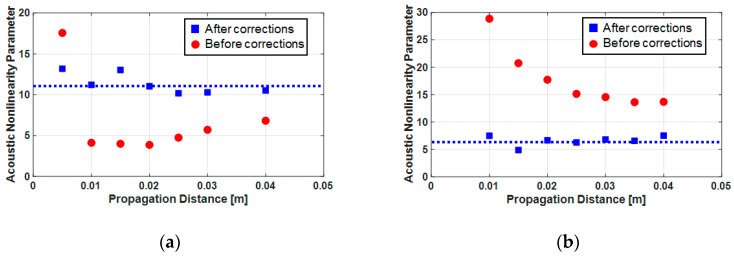
Absolute acoustic nonlinearity parameter results before the corrections (red dots) and after the corrections (blue squares): (**a**) fused silica and (**b**) Al6061-T6.

Figure 16 shows the results of the absolute acoustic nonlinearity parameter obtained from the results in Figure 15. The red dots are before the correction and the blue squares are the results after the correction; the results after correction were significantly more stable than those before the correction. Table 1 compares the averaged values of the acoustic nonlinearity parameters for each propagation distance obtained after calibration with the values reported in the literature. The results of the fused silica were similar to those in the literature while those of the Al6061-T6 were slightly higher than in the literature. However, the latter could be considered reasonable considering the deviation of the measured values of the nonlinearity parameter of Al6061-T6 reported in the literature.

## 7. Conclusions

In this study, a method to compensate the effect of the incident second harmonic component in an experimentally obtained second harmonic displacement amplitude was proposed for the precise measurement of the absolute acoustic nonlinearity parameter using a novel calibration method.

For this, the second harmonic component detected by the receiving transducer was considered as the sum of the component due to material nonlinearity and the component due to the incident signal and a numerical calculation model considering diffraction was developed as a function of the propagation distance. A linear wave propagation model was applied for the fundamental wave and the incident second harmonic and a nonlinear generation model in the quasi-linear approximation was applied to the component due to material nonlinearity. In the model, the factors related to the material nonlinear parameter and the magnitude of the incident second harmonic were unknown; these were determined by finding a value that best matched the experimental data according to the change in the propagation distance and then compensation for the incident second harmonic component was achieved. In particular, the case where the phase of the second harmonic due to material nonlinearity was opposite to that of the fundamental wave was also considered.

In the experiment to validate the developed method, fused silica and Al6061-T6 specimens with different thicknesses were tested. The choice of the two different materials was for testing both cases where the second harmonic due to material nonlinearity was in phase as well as out of phase with the fundamental component. The different thicknesses of the specimen were to change the propagation distance. The experimental results showed that the nonlinear parameters changed significantly according to the propagation distance before compensation but were very stable after compensation in both materials. The average values of the nonlinear parameter were also 11.04 in the fused silica and 6.59 in the Al6061-T6, both of which agreed well with the literature values. From these results, the proposed compensation method was effective at removing the incident second harmonic component in the experimental data. It should be noted that the attenuation effect was not considered in the numerical model because the fused silica and Al6061-T6 tested in this study had low attenuation. In cases where a significant attenuation effect exists, such as steel, the attenuation terms must be considered in the theoretical results. In a further study we will widen the scope of applicable materials by deriving a new theoretical model with the attenuation effect added.

## Figures and Tables

**Table 1:** Absolute acoustic nonlinearity values of this study and key references.

	This Study	Ref. 1 [7]	Ref. 2 [27]	Ref. 3 [29]	Ref. 4 [30]	Ref. 5 [31]
Fused silica	11.04	12.4	10.1	10.1		
Al6061-T6	6.59	4.5	5.41		5.69	6.12

## Data Availability

The data presented in this study are available on request from the corresponding author.
